# Treatment of depression in the elderly with repetitive transcranial magnetic stimulation using theta-burst stimulation: Study protocol for a randomized, double-blind, controlled trial

**DOI:** 10.3389/fnhum.2022.941981

**Published:** 2022-08-31

**Authors:** Leandro Valiengo, Bianca S. Pinto, Kalian A. P. Marinho, Leonardo A. Santos, Luara C. Tort, Rafael G. Benatti, Bruna B. Teixeira, Cristiane S. Miranda, Henriette B. Cardeal, Paulo J. C. Suen, Julia C. Loureiro, Renata A. R. Vaughan, Roberta A. M. P. F. Dini Mattar, Maíra Lessa, Pedro S. Oliveira, Valquíria A. Silva, Wagner Farid Gattaz, André R. Brunoni, Orestes Vicente Forlenza

**Affiliations:** ^1^Interdisciplinary Neuromodulation Service (SIN), Department and Institute of Psychiatry, Hospital das Clínicas HCFMUSP, Faculty of Medicine, Universidade de São Paulo, São Paulo, Brazil; ^2^Laboratório de Neurociências (LIM-27), Departamento e Instituto de Psiquiatria, Hospital das Clínicas HCFMUSP, Faculdade de Medicina, Universidade de São Paulo, São Paulo, Brazil; ^3^Programa de Fisiopatologia Experimental, Faculdade de Medicina, Universidade de São Paulo, São Paulo, Brazil

**Keywords:** depression, major depressive disorder, elderly, antidepressant, repetitive transcranial magnetic stimulation (rTMS), theta-burst stimulation (TBS), neuromodulation

## Abstract

**Introduction:**

Transcranial magnetic stimulation (TMS) is a consolidated procedure for the treatment of depression, with several meta-analyses demonstrating its efficacy. Theta-burst stimulation (TBS) is a modification of TMS with similar efficacy and shorter session duration. The geriatric population has many comorbidities and a high prevalence of depression, but few clinical trials are conducted specifically for this age group. TBS could be an option in this population, offering the advantages of few side effects and no pharmacological interactions. Therefore, our aim is to investigate the efficacy of TBS in geriatric depression.

**Clinical trial registration:**

[https://clinicaltrials.gov/ct2/], identifier [NCT04842929].

## Introduction

Major depressive disorder (MDD) is a problem among the elderly, with a prevalence of 31.74% (Zenebe et al., 2021). Several studies have demonstrated that MDD worsens the prognosis of cardiovascular and cerebrovascular diseases, the main causes of morbidity and mortality in the elderly (Horsten et al., 2000; Abramson et al., 2001; Matthews et al., 2010; Yary et al., 2010). In addition, clinical trials in patients with cardiovascular disease have demonstrated that antidepressant use decreases mortality (Taylor et al., 2005), whereas the severity and persistence of depression in patients with an acute myocardial infarction have increased mortality over subsequent years (Jiang et al., 2011). The presence of MDD is also associated with decreased cognitive and functional capacity (Ancelin et al., 2010). Thus, the treatment of depression in the geriatric population is important not only from a psychiatric point of view, but also has clinical and functional relevance.

The treatment of choice for geriatric unipolar depression is antidepressants. The therapeutic response is considered to be similar to adult depression, with remission rates that stabilize at 60–70% after more than three antidepressant treatments; therefore, approximately 30% of patients are resistant to pharmacological interventions (Rush et al., 2006). However, few clinical trials have focused specifically on the elderly population; much of the evidence for elderly antidepressant drug therapy is extrapolated from adult clinical trials or *post-hoc* analyses of these trials focusing on the elderly subgroup (Giron et al., 2005). Interestingly, a clinical trial that recruited patients >75 years of age did not demonstrate superior efficacy of citalopram over placebo in the treatment of depression (Roose et al., 2004). Yet, in pharmacotherapy for geriatric depression, important issues are the side effects, drug interactions, and restrictions on the use of antidepressants in this age group. For example, the elderly are more sensitive to the anticholinergic effects (constipation, urinary retention) characteristic of tricyclic antidepressants and the noradrenergic (dry mouth, tremor) effects of dual inhibitors, as well as the hyponatremia induced by selective serotonin reuptake inhibitors (Stahl and Stahl, 2013; Grover et al., 2018). Medications for therapeutic potentiation of antidepressants also have important side effects, such as intoxication by decreased renal clearance and dehydration with lithium and weight gain and extrapyramidal symptoms with antipsychotics (Stahl and Stahl, 2013). For these reasons, in this age group, the therapeutic adherence to pharmacological treatment for depression is usually low (Sanglier et al., 2011). Among the biological treatments, we highlight electroconvulsive therapy (ECT) and repetitive transcranial magnetic stimulation (rTMS). Both treatments are approved for depression and effective in the geriatric population (Salzman et al., 2002; Pallanti et al., 2012), but they have some issues that limit their use. In some cases, ECT is associated with long-term cognitive deficits, and its application requires sedation. As such, ECT is usually reserved only for cases of severe, refractory, and/or psychotic depression (Brunoni et al., 2010). In contrast, rTMS is a consolidated treatment for depression with several meta-analyses demonstrating its efficacy (Slotema et al., 2010; Chen et al., 2017). However, few studies have specifically evaluated the efficacy of rTMS in the geriatric population (Manes et al., 2001; Mosimann et al., 2004; Nahas et al., 2004; Sayar et al., 2013; Qin et al., 2017), though rTMS may have some advantages over medications for the treatment of MDD in the elderly population. First, rTMS offers few side effects, with headache being the most common complaint (Machii et al., 2006). The most serious adverse event is a seizure; however, it is an extremely rare event due to safety parameters, with few reports of cases in the literature (Miniussi et al., 2013). In addition, the risk of drug interactions observed with antidepressants and other continuous use medications is null, a situation very common among depressed elderly people. Furthermore, rTMS does not negatively interfere with patients’ cognitive function, and may even improve it to some extent (Sanna et al., 2019). Theta-burst stimulation (TBS) is an rTMS method that more closely mimics the natural activity rhythms in the neurons of the brain and uses short bursts of stimulation at high frequencies, with the bursts themselves being applied 5 times per second (Blumberger et al., 2018). Its main advantage over conventional rTMS is the shorter session duration (3–12 min vs. 20–30 min). One non-inferiority trial showed that TBS had similar efficacy as conventional rTMS (Blumberger et al., 2018). No controlled studies have been performed of the treatment of geriatric depression with TBS, but recent studies of the method have shown good prospects for the treatment of major depression in adults, with meta-analyses demonstrating potential clinical superiority over conventional rTMS (Duprat et al., 2016; Brunoni et al., 2017). In addition, the time for which the patient stays in the machine during TBS is much shorter, which facilitates patient compliance.

We want to evaluate whether TBS is a therapeutically effective alternative with few side effects in the geriatric population with depression. Therefore, we will conduct a parallel-group, randomized, controlled, clinical trial comparing active vs. sham TBS. This may result in short-term clinical gains for depressed elderly patients who do not tolerate antidepressants or have been refractory to antidepressants.

## Methods/design

### Design and population

The study will be a randomized, double-blind, sham-controlled clinical trial in which volunteers will be recruited at the Clinical Hospital of the Medical School of the University of São Paulo. They will be allocated to one of two groups: active TBS or sham stimulation. Participants will receive 20 consecutive work days of TBS and will return at the end of 6, 8, and 12 weeks for a single session and evaluation of clinical outcomes. Those who do not present clinical improvement and were allocated to the sham group may choose to receive 20 days of active stimulation after the end of the trial. The study is ongoing (start date January, 2019); first enrollment occurred in April, 2020 and only 20% of the sample has been collected thus far. We will use advertisements on social media to recruit additional volunteers outside the hospital.

### Randomization and allocation of participants

The randomization will be performed in blocks with permutation of the order and size of the blocks. The randomization will be generated using an independent website^1^ by a researcher who will not be directly linked to the research. Patient allocation will be carried out using sealed, opaque, and standardized envelopes. After the patient signs the informed consent form, an envelope will be opened and treatment allocated in a coded form. Each envelope will be identified by a random number that is assigned to each patient. One person external to the study in regard to the evaluation of patients and the data will be responsible for this part. Investigators and staff will make phone calls and send emails to volunteers to remind them of the upcoming trial dates to promote participant retention.

### Inclusion and exclusion criteria

Patients older than 60 years of age with initial Hamilton Depression Scale (HDRS) rating >17 and a diagnosis of MDD will be recruited for the study. The exclusion criteria will be other mental disorders (alcohol or drug addiction, psychotic disorders, dementia, bipolar disorder); the presence of serious neurological or clinical diseases; the presence of severe suicidal ideation; or Cumulative Illness Rating Scale (CIRS) score >7, characterizing a set of clinical morbidities that could impair adherence to the research protocol. In addition, specific contraindications to the use of rTMS will result in participant exclusion, such as metal implants, epilepsy, or electronics in the cephalic segment. Patients who are taking antidepressants will have a wash-out of five half-lives of the medication prior to entry into the study. Patients receiving benzodiazepines will be included in the study only when taking a maximum dose of 10 mg of diazepam or equivalent. Other psychoactive drugs will be accepted if the doses of the medications have been stable for at least 6 weeks. The participants can not have any change to or addition of any psychotropic with antidepressant effects during the trial; if this happens, they will be excluded from the trial.

### Blinding

The study will be double-blind; evaluators and patients will not be aware of the randomized treatment until the end of the study. The blinding will be done with a coil that is active and sham depending on the side used [Cool-B65 A/P, butterfly (figure 8) coil, active cooling, Magventure, Farum, Denmark], it is a symmetrical design with no indication of the active versus placebo side, with an adjustable output for current stimulation of the patient’s skin synchronous with the magnetic stimulation pulses, mimicking an active stimulation *via* a coil reproducing the sound that the true coil makes without generating the magnetic field. The person applying transcranial magnetic stimulation (TMS) will also be blinded; after placing the coil over the patient’s head and entering his number, the device notes whether the position is correct or if you have to turn it, but does not indicate whether it is the sham side of the coil. We will also place head electrodes that provide a stimulus to mimic the real magnetic coil (both groups receive this). A person meant to apply the rTMS will perform the procedure and not be involved with the evaluations or the evaluators, but this person will also be blinded. A blinding scale will be applied to evaluators, applicators, and volunteers to evaluate whether the blinding was effective.

### Interventions

The coil will be positioned in the prefrontal dorsolateral cortex, with intermittent TBS (iTBS) mode on the left and continuous TBS (cTBS) on the right (F3 and F4 positions determined by the 10–20 Electroencephalographic International System). We chose bilateral TMS based on the results of a network meta-analysis that suggested using bilateral and priming TMS for treating depression over conventional unilateral stimulation (Brunoni et al., 2017). There is no rationale for using cTBS first. A total of 1,800 pulses will be used on each side, for a total duration of 11 min and 39 s. TBS will have the following parameters: cTBS, bursts of three pulses at 50 Hz (20-ms interval between stimuli) will be applied continuously for 120 s total in the right DLPFC; iTBS, bursts of three pulses at 50 Hz (20-ms interval between stimuli) applied for 2-s duration, repeated every 10 s for a total of 570 s, also totaling 1,800 pulses in the left DLPFC, both with 120% motor threshold as used by Blumberger et al. (2018). The motor threshold will be measured every day prior to TMS with visualization of any motor twitch in the hand. We will use a magnetic stimulator device (MagVenture–Magpro X100) in study mode for double-blind trials. One side of the coil is active and the other is a sham. There will be 20 consecutive sessions, one per day, except on weekends and holidays, totaling approximately 4 weeks. A day of stimulation with the same technique will be performed in weeks 6, 8, and 12. The participants who do not complete the 20 sessions will be considered as drop-outs.

### Clinical variables

We will use the MINI questionnaire for the diagnosis of psychiatric disorders, and it will be applied by an experienced psychiatrist. It has been translated and validated in the Portuguese language (Sheehan et al., 1998). The demographic and clinical profiles of the patients will be evaluated by gender, age, schooling, socioeconomic status, clinical comorbidities, and use and dose of antidepressants and other psychoactive drugs. The main outcome will be measured using the HDRS scale. The primary evaluation will be performed at the end of 6 weeks from the start of the first day of TBS. As secondary outcomes, we will use the Geriatric Depression Scale (GDS), CIRS, global clinical impression scale (CGI), Positive and Negative Affect Scale (PANAS), Montgomery-Asberg Depression Rating Scale (MADRS), and Edinburgh Handedness Inventory (EHI). All scales have already been translated and validated in Portuguese (Gorenstein et al., 2000). Outcomes will be measured on seven occasions: baseline and 1, 2, 4, 6, 8, and 12 weeks after study entry. The presence of adverse effects and the Young mania (YMRS) questionnaire will also be evaluated to detect manic and/or hypomanic cycling. The first will be assessed by a questionnaire with eight questions consisting of yes/no answers regarding headache, cervical pain, hearing problems, cognition alterations, mood alterations, sleep alterations, nausea/vomiting, and other symptoms.

### Cognitive assessments

The cognitive battery will be applied by experienced neuropsychologists. All cognitive assessments will be measured at baseline and week 4, and the Addenbrooke Cognitive Examination will also be re-applied in week 12. We will use the Addenbrooke’s Cognitive Examination–Revised Version (ACE-R), Ravlt–Word List, Rey’s complex figure, Stroop Test (violet version), Wechsler Adult Intelligence Scale (WAIS III)–Subtest Codes, Trail Making A and B, Wechsler Abbreviated Scale of Intelligence (WASI)–Vocabulary and Matrix Reasoning subtest, and a computerized N-back test.

### Biomarkers

Samples of blood will be collected at the beginning of the study and in week 6 to measure brain-derived neurotrophic factor (BDNF). Cerebrospinal fluid (CSF) samples will be collected *via* lumbar puncture during the evaluation before intervention begins. They will be used to investigate beta-amyloid peptide (Aβ1-42) and Tau protein (total and phosphorylated). CSF Aβ42, t-tau/Aβ42, and p-tau/Aβ42 ratios are associated with neuropsychiatric symptoms (including depression) in community-dwelling individuals free of dementia (Krell-Roesch et al., 2022). We want to assess whether these Alzheimer’s biomarkers in the CSF predict a poorer response to TMS.

Assessment of sensitivity thresholds according to the Quantitative Sensitivity Test (QST) is performed using the psychophysical method that quantifies the positive and negative phenomena of exteroceptive sensitivity transmitted by the fine or thick fibers of the peripheral nervous system. Diffuse nociceptive inhibitory control (DNIC) or conditioned pain modulation (CPM) are terms used to demonstrate a reduction in pain in response to the application of painful stimuli outside the area of pain. Testing will be done prior to the start of the TBS sessions and in 4th week. QST, DNIC, and CPM correlate with pain threshold (Yarnitsky, 2010; Weaver et al., 2021), and we know pain is associated with depression (Sheng et al., 2017). Therefore, the objective of testing these parameters is to correlate them with changes in depression scores.

The pupil light response (PLR) will be assessed as the difference in the diameter of the pupil before and after a red flash of light. The participant will perform a test lasting approximately 20 min during which a camera phone will record the size of the pupil while the participant listens to some sounds. The exam is painless and without side effects. It will be performed at baseline and in week 4. Patients with depression have a significantly reduced transient pupillary response to low-intensity red light (Laurenzo et al., 2016), and we want to know if this correlates with the antidepressant response to TMS. On the day prior to randomization and initiation of treatment, an initial rTMS session will be carried out to assess the modulation of left MC excitability by iTBS. For this purpose, MEPs will be induced using single TMS pulses delivered to the MC at an intensity of 120% RMT, with a random stimulus interval of approximately 10 s (±1 s). Muscle relaxation will be monitored through visual inspection of the electromyographic signal (EMG) signal to ensure that single pulses are delivered in the absence of active muscle contraction. Motor evoked potential (MEP) amplitude will be measured peak-to-peak and averaged across 30 consecutive MEPs. Our group previously used the same paradigm in other populations, including those with a high risk of seizures, such as brain tumors and schizophrenia, without any seizures being induced (Gordon et al., 2019; dos Santos et al., 2021). Patients will then receive a single iTBS session over the left-hand muscle “hotspot” with the same parameters as those used for iTBS treatment. Approximately 30 s after completion of MC iTBS, MEP amplitude will be measured again in the same manner as prior to iTBS. An index of the modulation of MC excitability will be calculated as the percentage change in the mean MEP amplitude post-iTBS relative to pre-iTBS, with positive values (MEP amplitude increase) reflecting facilitation of cortical excitability by iTBS and negative values (MEP amplitude decrease) representing suppression. All of these parameters will be used as predictors of the treatment response as well as to determine neurophysiological changes before and after the treatment.

### Sample size calculation

Based on Li’s clinical trial (Li et al., 2014), which demonstrated an efficacy favoring bilateral TBS in depression (52.5% for the active group and 17.4% for the sham group), for a two-tailed *p*-value of 0.05 and a power of 90%, the total sample size would need to be 86 subjects. Considering a drop-out rate of approximately 25%, we estimated a final sample size of 108 patients.

### Statistical analysis and data management

All of the data will be electronically stored in RedCap software and checked weekly to ensure all data are adequately recorded. Statistical analyses will be performed in the Stata 17 ME program for Mac OS X. All analyses will be performed based on the intention to treat principle (i.e., the data from all participants will be included in the analysis), in which lost data will be allocated according to the last observation carried forward and/or mixed data. Analyses will be considered significant at *p* < 0.05. We will use parametric tests to analyze the main outcomes, which are allowed by the sample size. Number of previous admissions, presence of clinical comorbidities, and refractoriness (measured by the resistance to two antidepressants) will be analyzed as ordinal data. For the main outcome, we will use a repeated measures analysis with HDRS score as a dependent variable and TBS as an independent variable at week 8. Secondary analyses of the other scales and biomarkers will be performed in the same way, replacing HDRS as the dependent variable.

### Study flow chart

Table 1 shows the flow of each patient through the study. Note that TBS will be applied 23 times: 20 consecutive sessions (week 0 through week 4), and one session each in weeks 6, 8, and 12. Patients who receive sham stimulation and who are still depressed may receive active stimulation at the end of the study.

**TABLE 1:** Study schedule.

	Screening	Baseline	Week						
			
			1	2	3	4	6	8	12
Assessment of eligibility	X								
Structured interview (MINI)	X								
Consent form	X								
Theta-burst stimulation (active/sham)			D	D	D	D	W	W	W
Clinical interviews	X		X	X		X	X	X	X
Adverse effects			X	X		X	X	X	X
Blood collection for serum biomarkers		X					X		
Neuropsychological evaluation		X					X		X
Cerebrospinal fluid collection		X							

D, daily; W, weekly.

### Ethical aspects, safety, and dissemination

The study has been approved by the Research Ethics Committee (CAAE: 80215117.5.0000.0068). All enrolled subjects will consent to participate *via* an informed consent form. All of the described procedures present minimal risk. If a volunteer presents a risk of major suicide, they will be excluded from the study, adopting the standard procedure for the management of this type of patient. Participants may have access to their data and may leave the study at any time without prejudice to any treatment they may receive within the institution. The data will be collected, analyzed, and published in order to preserve the anonymity of the individual. In addition, the study will be conducted in accordance with all requirements of the Research Ethics Committee and based on the recommendations established in the Declaration of Helsinki (1964), as amended in Tokyo (1975), Venice (1983), and Hong Kong (1989). Participants will also be able to participate in a clinical trial to treat their clinical condition. This will be possible even if they receive sham stimulation, as they may receive active stimulation at the end of the study if they still have severe depressive symptoms. After completion, the study will be presented at congresses in the area and will be published in indexed journals. For any protocol modification, we will first ask the CEP, including additional consent for use of data and biological specimens not included in the informed consent in ancillary studies. We have made one amendment (February, 2020) to the protocol with the approval of the ethics committee: inclusion of the measurement of secondary outcomes QST, DNIC, CPM, and PLR. We decided to not have a data monitoring committee because rTMS is very safe when used properly and has few adverse effects, the most common being headache. Applications will always be performed in a hospital setting with a physician present. We will evaluate patients 23 times over 12 weeks so that we can quickly identify any worsening of the clinical picture and provide early intervention. All of the collected data will be collected, maintained, and analyzed only by the investigators of the project (as the final dataset), assuring the confidentiality of the volunteers. Although very low, the most serious side effect of TMS is induction of seizures. If this occurs, our lab is inside a tertiary hospital with an intensive care unit and protocols for seizures and epileptic status that can be used if necessary. The other risk is that we have a sham group not using antidepressants. The main risk is a high suicide risk. As the volunteers come to the hospital every day, any sign of the depression symptoms worsening will be evaluated by psychiatrists and, if the risk of suicide is high, the volunteer will be withdrawn from the study and given the appropriate treatment and management for the situation. In the wash-out phase, the volunteers will be evaluated at least once a week to observe symptoms for any worsening. If a high risk of suicide appears, we will re-start the treatment and the volunteer be withdrawn from the study.

## Discussion

Here, we provided the study protocol for a randomized controlled trial (RCT) that will evaluate the efficacy of TBS in the treatment of geriatric depression. To the best of our knowledge, this is one of the first studies in this field. The participants will receive 23 sessions of bilateral TBS, comprising first inhibitory stimulation delivered by cTBS in the right DLPFC followed by excitatory stimulation delivered by iTBS in the left DLPFC, totalizing 3,600 pulses. We performed a sample size calculation based on the effect size found in the most similar clinical trial in the literature (Li et al., 2014). No specific treatments are yet available using TMS or Transcranial direct current stimulation (tDCS) for depression in the elderly. We will enroll patients with different degrees of refractoriness and use TBS as the only treatment for depression (the antidepressants will be washed out), which will significantly increase the external validity of our results. These data could also contribute to the analysis of some predictors of the treatment response.

We expect that TBS will be statistically superior to sham treatment for depression as assessed by the HDRS. Other expected secondary outcomes are TBS being superior to sham treatment for improvement of depressive symptoms by the GDS, MADRS, and CGI. Regarding serum markers, we expect that the active vs. sham TBS groups will have a significant increase in BDNF levels. From the neurocognitive point of view, we expect that both groups will perform similarly. Regarding the neurophysiological biomarkers QST, DNIC, and CPM, we expect that the volunteers will be less sensitive to the stimulus. We also expect an increased pupillary response to low-intensity light.

## Strengths and limitations

A strength of this protocol is that it is one of the first clinical trials addressing the efficacy of TBS for MDD in the elderly. If the efficacy is proven, it could be an alternative therapeutic option for the treatment of MDD in this population. Another positive point of our study is that we will collect several different types of biomarkers that could predict the response to antidepressants. A limitation of the study will be that our age limit (>60 years old) can limit the generalization of the data to the very old population. Moreover, as we are using a bilateral protocol for rTMS, we cannot assume efficacy for other interventions (e.g., unilateral rTMS). We will also have limitations due to the biomarkers. For example, the study was not powered for them, so they will be an exploratory analysis. The cognitive tests will have the problem of changes caused by practice effect due to the short time interval between the tests.

## Conclusion

This study protocol was developed to investigate the efficacy of TBS for the treatment of MDD in elderly patients using a randomized, sham-controlled design. The biomarker investigation will contribute to understanding the neurobiological mechanisms of depression and their relationship with treatment. This trial will contribute valuable information to the treatment of depression in the elderly.

## Author contributions

LV was responsible for initiating and managing the trial, conceiving and designing the assay, analyzing the data, and writing the manuscript. PS, WG, AB, and OF performed the statistical analysis. LS, RB, CM, JL, and ML were responsible for the clinical evaluation. LT, HC, RV, and RD were responsible for the neuropsychological evaluation. PO was responsible for the collection of licor. KM and BP were responsible for collecting cortical excitability, DNIC, and CPM. BP and BT were responsible for the pupil examination. VS was responsible for the application of rTMS. All authors contributed to read and approved the final manuscript.

## Footnotes

1
www.randomization.com
